# Evaluation of HoloLens Tracking and Depth Sensing for Indoor Mapping Applications

**DOI:** 10.3390/s20041021

**Published:** 2020-02-14

**Authors:** Patrick Hübner, Kate Clintworth, Qingyi Liu, Martin Weinmann, Sven Wursthorn

**Affiliations:** Institute of Photogrammetry and Remote Sensing, Karlsruhe Institute of Technology, 76128 Karlsruhe, Germany; kate.clintworth@student.kit.edu (K.C.); qingyi.liu@student.kit.edu (Q.L.); martin.weinmann@kit.edu (M.W.); sven.wursthorn@kit.edu (S.W.)

**Keywords:** indoor mapping, augmented reality, HoloLens, time-of-flight camera, depth camera, tracking

## Abstract

The Microsoft HoloLens is a head-worn mobile augmented reality device that is capable of mapping its direct environment in real-time as triangle meshes and localize itself within these three-dimensional meshes simultaneously. The device is equipped with a variety of sensors including four tracking cameras and a time-of-flight (ToF) range camera. Sensor images and their poses estimated by the built-in tracking system can be accessed by the user. This makes the HoloLens potentially interesting as an indoor mapping device. In this paper, we introduce the different sensors of the device and evaluate the complete system in respect of the task of mapping indoor environments. The overall quality of such a system depends mainly on the quality of the depth sensor together with its associated pose derived from the tracking system. For this purpose, we first evaluate the performance of the HoloLens depth sensor and its tracking system separately. Finally, we evaluate the overall system regarding its capability for mapping multi-room environments.

## 1. Introduction

The Microsoft HoloLens is a mobile, head-worn augmented reality (AR) device introduced in 2016. It is capable of augmenting the physical environment of the user with virtual content, called ’holograms’, rendered into its transparent stereoscopic display unit. Meanwhile, the device is widely used in different application areas like human-robot interaction [1,2], industrial process management [3] and engineering [4], facility management [5], surgery [6] or education [7,8].

For a satisfying AR experience, a stable registration of virtual content relative to the physical surrounding of the user is of utmost importance. To achieve this, the HoloLens is equipped with a variety of sensors including four tracking cameras and a time-of-flight (ToF) range camera. Localization and mapping of indoor spaces are done directly on the device in real-time in a SLAM-like manner. In this context, range measurements are aggregated in the form of triangle meshes representing the physical environment in which the device is operating. Knowledge about the geometric structure of its surrounding allows for a reasonable placement of virtual content and enables a realistic interaction of holograms with the real world. These resulting triangle meshes as well as range images and their poses estimated by the built-in tracking system can be accessed by the user. This makes the HoloLens potentially interesting as an indoor mapping device.

In this work, we provide a comprehensive evaluation of the Microsoft HoloLens (Version 1) regarding its adequacy for the task of the three-dimensional mapping of indoor environments. This comprises independent evaluations of the depth sensing and tracking capability of the device against ground truth as these together constitute the mapping performance. Furthermore, point clouds resulting from depth sensing data and poses from the tracking system as well as the preprocessed triangle meshes are evaluated to assess the indoor mapping capability of the system at large.

The HoloLens has already been evaluated in regard of a range of different aspects. For instance, Liu et al. [9] provide a first basic evaluation of the HoloLens as AR device, while Vassallo et al. [10] specifically investigate the perceived spatial stability of holograms. An investigation regarding the quality of the usage experience enabled by the HoloLens is provided by Zhang et al. [11]. Kirks et al. [12] evaluate the HoloLens tracking system against ground truth data from a motion capture system for application scenarios in the field of human-robot interaction. Hübner et al. [13] and Khoshelham et al. [14] present first quantitative investigations on the overall spatial accuracy of triangle meshes captured by the HoloLens against ground truth data from terrestrial lasers scanners (TLS) for the use-case of indoor mapping. However, to the best of our knowledge, there is no published work so far directly evaluating the ToF range sensor of the device instead of the triangle meshes derived from it.

Seminal work in regard of the evaluation of depth cameras includes for example the work of Khoshelham and Oude Elberink [15], where the focus is set on evaluating the widely-used first version of the Microsoft Kinect depth camera which however is not a ToF sensor, but a sensor relying of structured light projection. Weinmann et al. [16] provide a comparative evaluation of the Kinect in relation to a ToF depth camera. Later work evaluating ToF sensors includes e.g., the investigations of Gonzalez-Jorge et al. [17], Lachat et al. [18], Sarbolandi et al. [19] or Fürsattel et al. [20].

A well-known metric for the evaluation of mobile inside-out tracking systems is the absolute trajectory error and relative pose error proposed by Sturm et al. [21] which we also rely upon in this work in the scope of performance evaluation.

Notable work concerning the evaluation of mobile indoor mapping systems encompasses for example the work of Lehtola et al. [22] who provide a comparative evaluation of a variety of different indoor mapping systems, while Chen et al. [23] comparatively evaluate SLAM-based indoor mapping systems. Two typical off-the-shelf mobile mapping systems are compared by Nocerino et al. [24] and evaluated against ground truth data in outdoor as well as indoor settings. Masiero et al. [25] compare a photogrammetry-based indoor mapping system with one based on laser scanners. Blaser et al. [26] present evaluation results of their own mobile mapping system based on a panorama camera and laser scanners, while Lagüela et al. [27] present a mobile indoor mapping system based on a LiDAR sensor resulting in comparatively sparse point clouds. An evaluation procedure for indoor point clouds in the absence of ground truth data is presented by Karam et al. [28].

To the best of our knowledge, this paper presents the first evaluation of the HoloLens depth sensing capability based not on the pre-processed coarse triangle meshes but on the range images as raw output of its ToF depth sensor. While the HoloLens has been investigated before regarding its aptitude to the task of indoor mapping, previous work on this topic focused on the examination of the results, i.e., triangle meshes of indoor environments and their accuracy. Beyond evaluating indoor mapping results in the form of triangle meshes and point clouds, we comprehensively evaluate the whole system by additionally investigating the depth sensor and the tracking system separately as these two subsystems mainly constitute the performance of the overall system in regard to the task of indoor mapping. In doing so, we show that while the HoloLens clearly holds potential for efficiently capturing the three-dimensional geometry of indoor environments, its system is tailored towards its primary use-case as an indoor AR device. Thus, its capabilities in tracking and sensing its surrounding are sufficient for providing locally consistent results that enable a high quality of convincing AR experience. However, there are shortcomings in the form of drift effects on larger scales excelling the ordinary needs of an AR system.

In the following section, we commence by briefly specifying the different sensors the HoloLens is equipped with and their respective characteristics in Section 2.1. Subsequently, we elaborate on the details of our evaluation procedures in the rest of Section 2, before we present the results of the conducted evaluation in Section 3. In this context, we first focus on the evaluation of the HoloLens ToF range sensor, then proceed with the evaluation of the HoloLens tracking system and finally conclude with the evaluation of the HoloLens system at large regarding its adequacy for the mapping of indoor environments. The achieved results are discussed in detail in Section 4. Finally, we provide concluding remarks in Section 5.

## 2. Materials and Methods

**Figure 1:**
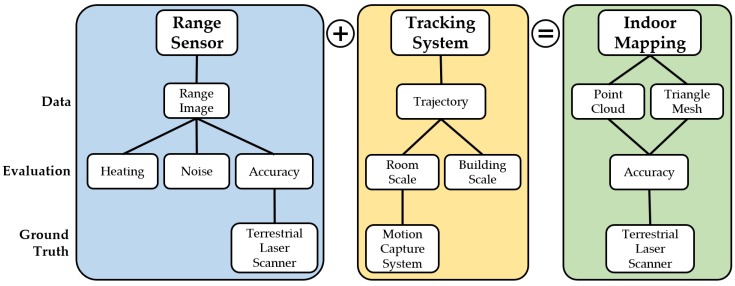
Schematic overview of the evaluation of the Microsoft HoloLens as indoor mapping system with range sensor and tracking system as the relevant subsystems constituting it.

In this section, we describe the experiments conducted for assessing the capabilities of the Microsoft HoloLens in regard to its aptitude for the task of indoor mapping. Section 2.1 gives an overview of the different camera sensors the device is equipped with and their respective characteristics. Afterwards in Section 2.2, we comprehensively elaborate on the evaluation procedures of the different experiments conducted in the course of this study. A schematic overview of the whole evaluation procedure is depicted in Figure 1.

### 2.1. Sensor Description

**Figure 2:**
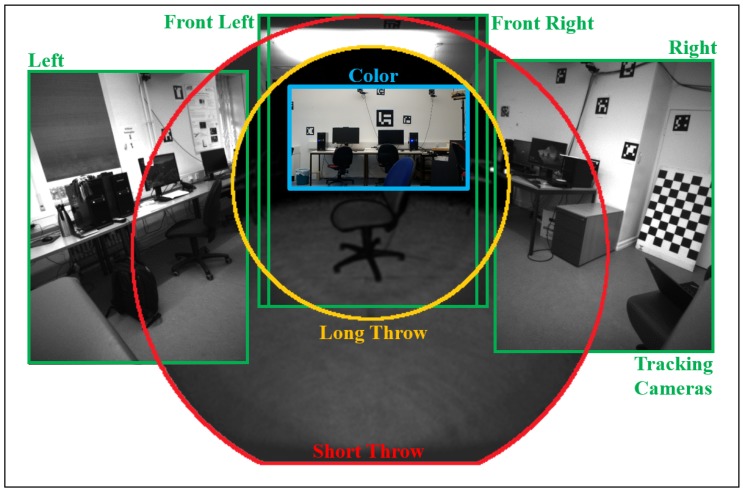
Overlay of images recorded by the different camera sensors of the Microsoft HoloLens as specified in Table 1.

The Microsoft HoloLens device is equipped with various imaging sensors providing data necessary to accomplish the different tasks constituting its mobile indoor augmented reality system such as tracking, re-localization in known environments and capturing the geometric structure of its surroundings by means of depth sensing. Table 1 gives an overview of these camera sensors and their respective characteristics, while Figure 2 shows an overlay of images recorded by those sensors to give an impression about their arrangement on the device.

All camera sensors of the HoloLens can be queried via the Microsoft Windows 10 SDK [29]. However, for all cameras except for the color camera, a so-called ’research mode’ has to be activated. This mode is only meant for research. Applications making use of it cannot be used in apps published on the Microsoft Store for applications.

The color camera can be queried in different resolutions. It is not used for tracking, but only for allowing the user to record screenshot videos and pictures. Virtual renderings augmenting the physical environment of the user wearing the device can optionally be rendered into the images captured with this camera. The center of the color image in Figure 2 roughly aligns with the line-of-sight of the user wearing the device.

Besides this color camera, the device also includes four grayscale tracking cameras, two of which are oriented to the front in a stereo configuration with large overlap, while the other two are oriented to the right and left respectively with nearly no overlap to the center pair as depicted in Figure 2. The images of these tracking cameras are provided by the SDK as rotated by 90°, but their attached poses correct for this rotation. It is worth mentioning that the SDK returns 160 × 480 4-channel 8-bit images when querying the grayscale tracking cameras. These images actually represent 640 × 480 1-channel grayscale images where the intensity values are spread line-wise over all 4 channels. So the first pixel of the first line of the 160 × 480 image contains the first 4 pixels of the first line of the 640 × 480 image in its 4 channels.

The HoloLens device is furthermore equipped with a time-of-flight (ToF) depth sensing camera, providing images with pixel-wise range measurements. These range images can be queried by the SDK in two different modes, termed ’long throw’ and ’short throw’. Short throw data contain distance values in the range of 0 m to 0.8 m, while long throw data contain distance values from 0.8 m to about 3.5 m. For both modes, depth sensing data is delivered by the SDK in the form of 16-bit range images where the pixels contain integer values representing distance in millimeters. Furthermore, 8-bit grayscale images representing infrared reflectivity can be queried for both modes.

**Figure 3:**
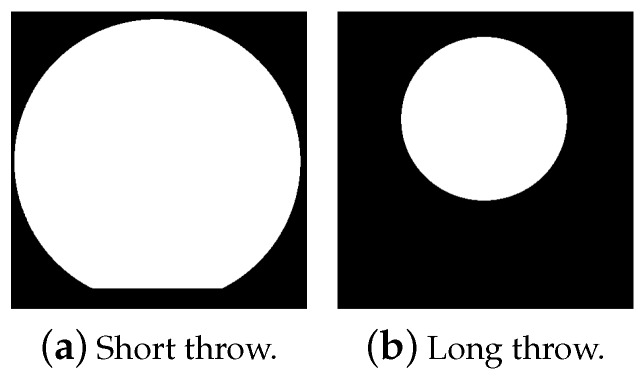
Pixels actually containing values are shown in white for short throw and long throw mode.

All images acquired by the ToF sensor have a size of 448 × 450 pixels; however different parts of the images actually contain values as depicted in Figure 3. The part of the image actually containing values is circular for both modes. In the case of the long throw mode, this circular area containing range measurements is bigger and slightly clipped on the lower side.

The depth sensing camera is oriented slightly downwards relative to the line-of-sight of the user as can be seen in Figure 2. In typical usage scenarios, the short throw mode mainly observes the hands of the user for gesture recognition, while long throw range data are used for environment mapping. The field-of-view of the ToF camera overlaps with the one of the color camera; however the color image covers only a fraction of the range images.

The inner orientations of all camera sensors can be queried from the SDK in the form of a matrix, mapping from pixel coordinates $\begin{array}{cc} (x & y) \end{array}$ to metric 2D coordinates $\begin{array}{cc} (U & V) \end{array}$ on a plane in 1 m distance from the respective camera. An inverse mapping is also provided.

The range images delivered by the ToF sensor contain distance values along rays through the respective point $\begin{array}{cc} (U & V) \end{array}$ on the unit plane for each pixel $\begin{array}{cc} (x & y) \end{array}$. To transform these range values *R* to depth values *D* along an axis parallel to the image plane, the following equation can be used:

(1) $$D=\frac{R}{\sqrt{U^{2}+V^{2}+1}}$$

Thus the Cartesian coordinates of a 3D point $\begin{array}{ccc} (X & Y & Z) \end{array}$ can be derived:

(2) $$\left(\begin{array}{c} X\\ Y\\ Z \end{array}\right)=D\left(\begin{array}{c} U\\ V\\ 1 \end{array}\right)$$

**Figure 4:**
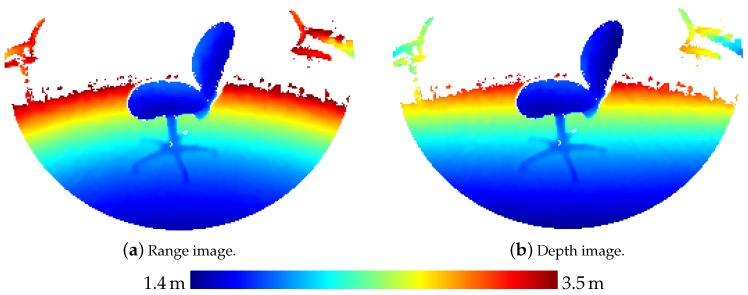
Range image and depth image corresponding to the long throw intensity image shown in Figure 2.

Figure 4 shows the range and depth image respectively corresponding to the reflectivity image for the long throw mode of the ToF camera shown in Figure 2.

For all images, the corresponding camera pose in a coordinate frame defined by the initial pose when starting the respective HoloLens application can be obtained from the SDK. However, the camera pose is provided as split in two relative poses. One of these poses ($T_{Device}^{Origin}$) consists only of a translational component and describes the position of the HoloLens device with constant orientation. The other pose is the inverse of the pose $T_{Camera}^{Device}$ of the respective camera relative to this first pose. The current orientation of the device is encompassed in this second pose that furthermore contains a translational component for the offset of the respective camera from the point of reference of the device pose. The absolute pose of the respective camera then results to

(3) $$T_{Camera}^{Origin}=T_{Device}^{Origin}T_{Camera}^{Device}$$

Besides raw range images captured by the ToF sensor, the HoloLens SDK also provides preprocessed triangle meshes derived from the range data. Usage of these triangle meshes is not restricted to the research mode.

### 2.2. Evaluation Method

To assess the adequacy of the Microsoft HoloLens for the usage as a mobile indoor mapping device, we conducted a range of experiments which are detailed in this section. First in Section 2.2.1 we describe the evaluation of the depth sensor of the HoloLens device. This is followed by the presentation the evaluation of its tracking system in Section 2.2.2, while we finally describe the evaluation of the combined system for the use-case of indoor mapping in Section 2.2.3.

#### 2.2.1. Depth Sensing

In our evaluation of the HoloLens depth sensing capability, we focus on the long throw mode mentioned in Section 2.1 as the short throw mode is only used for gesture recognition and thus not of relevance regarding the use-case of indoor mapping. For the conducted experiments, a plain, white, planar wall was used as reference object. The HoloLens device was fixed on a stand facing the wall and recording long throw range images.

First, we investigated the influence of the heating process on range measurements by capturing a static scene and analyzing the temporal variation of the resulting range data. The device was positioned in a distance of about 1 m, approximately perpendicular to the wall surface with the wall filling the whole field-of-view of the long throw range images. The recording of the data was started with a completely cooled-down device, several hours after its last usage. Range images were recorded for a duration of 100 min with a frame rate of 1 fps in four consecutive recordings with 25 min length each and pauses of only a few seconds in between. The reason for splitting the measurement is that the device switches to sleep mode after 30 min without movement. Subsequently, the change in the mean depth value resulting from the respective range images was analyzed to characterize the influence of the warm-up process of the device over time.

To assess the influence of the measured distance on sensor noise, we furthermore varied the distance of the sensor to the wall while keeping the sensor approximately perpendicular to the wall surface. In this manner, recordings of several minutes length each were made at varying distances over the whole working range of the sensor (0.8 m to about 3.5 m).

In the subsequent analysis, the standard deviation of the measured distance values per pixel was determined for each probed sensor position. Mean standard deviations over all pixels were then determined per sensor position, once for all pixels considered for evaluation and once only for those pixels that have recorded range values in at least 75% of the images of the respective recording.

In doing so, only pixels representing wall surface were considered for evaluation. In the cases where parts of floor, ceiling, lateral margins of the wall surface, etc. are visible, which happens with growing distance of the sensor from the wall, binary masks were created manually based on the reflectivity images for excluding those pixels not belonging to the wall surface from the evaluation.

Furthermore, the influence of the inclination of the wall surface on sensor noise was also investigated for inclinations between 0° and 80°. Here, the same analysis as in the above-mentioned evaluation of the influence of distance was conducted.

**Figure 5:**
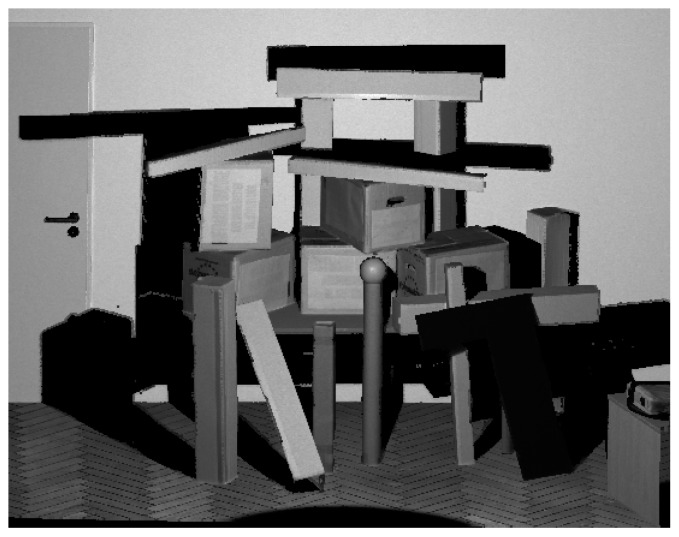
Ground truth data captured for a three-dimensional scene using a terrestrial laser scanner (Leica HDS 6000).

Besides those experiments where a flat wall surface was used as reference object, sensor noise was also investigated on a three-dimensional scene comprising simple, geometric bodies (boxes, cylinders and spheres) as depicted in Figure 5. This scene was captured by the HoloLens from three different distances.

Furthermore, the three-dimensional scene was also used for an evaluation of the accuracy of the captured distances using ground truth data acquired by a terrestrial laser scanner (Leica HDS 6000). To this aim, the point clouds derived from the range images were manually registered on the ground truth point cloud with a subsequent refinement of the registration via the Iterative Closest Point (ICP) algorithm [30,31]. Then, Euclidean distances to the nearest ground truth point were determined for each HoloLens point. Afterwards, the HoloLens points were transformed back to the pixel grid of the original range images for better visual interpretation.

For this analysis, the software Cloud Compare [32] was used. Furthermore, we make use of the software provided by Microsoft [33] for accessing range images with accompanying poses from the ToF sensor.

#### 2.2.2. Tracking

**Figure 6:**
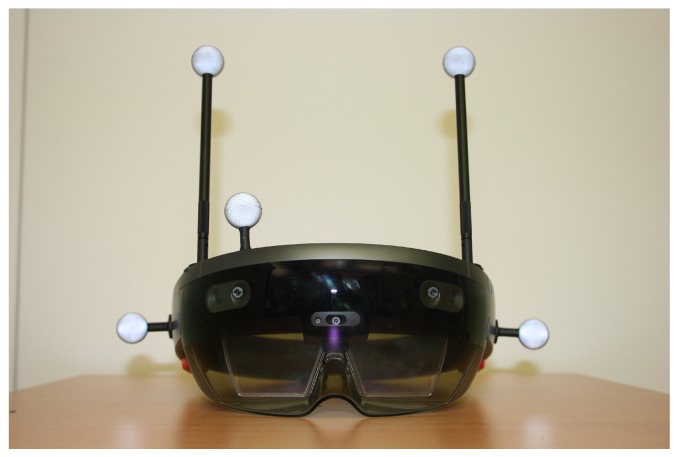
Rigid body affixed to the HoloLens device.

For assessing the tracking capacity of the HoloLens, the optical motion capture system OptiTrack Prime 17W [34] with eight tracking cameras in a laboratory room with a size of approximately 8 m × 5 m × 3 m was used to get ground truth data. For this purpose, the HoloLens device was equipped with a rigid body consisting of five reflecting sphere markers trackable by the motion capture system as depicted in Figure 6.

The spatial offset $T_{RigidBody}^{Device}$ between the local coordinate system constituted by those rigid body markers and the local HoloLens device coordinate frame, whose poses are recorded by the HoloLens tracking system, had to be determined by a calibration procedure. For this purpose, a checkerboard pattern was observed by the HoloLens color camera in a static setting, while the device was equipped with the rigid body. The pose $T_{Camera}^{Checkerboard}$ of the camera relative to the local coordinate system of the checkerboard was determined via the Perspective-n-Point (PnP) algorithm [35], while the relative pose $T_{Camera}^{Device}$ of the camera with respect to the local coordinate system of the HoloLens itself was acquired from the Windows 10 SDK.

By manually measuring the positions of the sphere markers of the rigid body and the corners of the checkerboard pattern with a tachymeter (Leica TS06), the poses of the checkerboard ($T_{Checkerboard}^{Tachymeter}$) and the rigid body ($T_{RigidBody}^{Tachymeter}$) in the local coordinate frame of the tachymeter were determined. The pose $T_{RigidBody}^{Device}$ of the rigid body in the local coordinate frame of the HoloLens device could thus be determined as:

(4) $$\begin{array}{cc} T_{RigidBody}^{Device} & =T_{Camera}^{Device}T_{Checkerboard}^{Camera}T_{Tachymeter}^{Checkerboard}T_{RigidBody}^{Tachymeter}\\ \; & =T_{Camera}^{Device}T_{Camera}^{Checkerboard^{-1}}T_{Checkerboard}^{Tachymeter^{-1}}T_{RigidBody}^{Tachymeter} \end{array}$$

Device trajectories of poses $T_{Device}^{HoloLensOrigin}$ acquired via the HoloLens tracking system could now be transformed to trajectories of poses $T_{RigidBody}^{HoloLensOrigin}$ of the rigid body attached to the device and thus be compared to the ground truth trajectories of poses $T_{RigidBody}^{MotionCaptureSystem}$ observed by the motion capture system:

(5) $$T_{RigidBody}^{HoloLensOrigin}=T_{Device}^{HoloLensOrigin}T_{RigidBody}^{Device}$$

A prevalent metric for the evaluation of estimated trajectories against ground truth trajectories is represented by the Absolute Trajectory Error (ATE) and the Relative Pose Error (RPE) [21].

For determining the ATE, it is essential to spatially align the trajectory with its corresponding ground truth trajectory when they are given in distinct coordinate frames as is the case here. Furthermore, a temporal alignment by timestamps $t_{i}$ between corresponding poses of both trajectories is required, that allows to assign each pose $P_{i}$ of the trajectory its temporarily closest ground truth pose $P_{i}^{GT}$.

As the poses acquired by the motion capture system only have timestamps relative to the start time of the measurement, a temporal alignment between the HoloLens trajectory and the trajectory of the motion capture system had to be conducted. This was achieved by manually extracting timestamps at trajectory positions on the apex of distinct peaks in the trajectories.

The thus temporarily assigned pose pairs $P_{i}$ and $P_{i}^{GT}$ could then be used to spatially align both trajectories by the method of Horn [36] as proposed by Sturm et al. [21] while keeping the scale fixed. With the trajectories registered in a common coordinate frame, the ATE could be calculated by the root mean square error

(6) $$ATE=\sqrt{\sum_{i=0}^{N}||trans\left(D_{i}\right)\left|\right|}$$

of the translational components of the pose differences $D_{i}$ between corresponding HoloLens and ground truth poses:

(7) $$D_{i}=P_{i}^{{GT}^{-1}}P_{i}$$

The ATE is only meaningful as an aggregated value like the root mean square error over a complete trajectory as the quantity of translational differences of particular pose pairs results from the alignment process between both trajectories and not from the tracking quality in the respective poses themselves. Thus, the ATE can only be regarded as a measure for tracking quality over the whole trajectory.

To eliminate the subjective influence of manually selecting a pose pair for temporal alignment between both trajectories, an optimization procedure is applied that determines the temporal alignment in millisecond-resolution by minimizing the ATE.

The RPE on the other hand is a metric for relative drift between an estimated trajectory and its ground truth trajectory. Like the ATE, it is calculated as root mean square error of the translational (or rotational) components of pose differences Equation (6). Here, however, the pose differences $D_{i}$ are relative differences based on an offset Δ in the pose index:

(8) $$D_{i}={\left(P_{i}^{{GT}^{-1}}P_{i+\Delta }^{GT}\right)}^{-1}\left(P_{i}^{-1}P_{i+\Delta }\right)$$

We applied as Δ the number of poses corresponding to the time difference of one second to get the RPE as a value for drift per second.

We evaluated the ATE and RPE metrics for trajectories recorded while walking around in the laboratory space covered by the cameras of the motion capture system while following the same pattern of movement for each trajectory. We varied the conditions by masking the depth camera for some of the recorded trajectories.

Furthermore, to assess the influence of drift on large-scale trajectories through long corridors in large building complexes, a trajectory with accompanying triangle meshes was recorded along a long closed loop of a total length of 287 m on two floors of a building. The trajectory ended in the same room it started in, while the room was re-entered through a different door than it was left through. The course of the trajectory was planned and executed in a way that ensures that the re-localization system of the HoloLens is only able to detect the drift-induced failure in its position and correct for it, when the device has already re-entered the room.

#### 2.2.3. Indoor Mapping

After the evaluation of the individual components relevant for indoor mapping, depth sensor and tracking system, we furthermore evaluated the performance of the overall HoloLens system in regard of indoor mapping. For this purpose, we mapped an indoor space of an office environment comprised of six rooms with furniture resulting in triangle meshes by the Spatial Mapping System of the HoloLens. For a subset of four of these rooms, range images in the long throw mode were also recorded. While the acquisition of the triangle meshes was conducted by walking through the rooms leisurely in typical walking speed, the acquisition of the range images was done by walking deliberately slow, as range images can currently only be acquired with a rate of one frame per second.

The range images were subsequently transformed to a global point cloud making use of the poses of the range images provided by the tracking system. This point cloud as well as the triangle meshes were manually registered on a ground truth point cloud of the mapped indoor environment acquired by a terrestrial laser scanner (Leica HDS 6000) with subsequent ICP-based refinement [30,31]. As the ground truth point cloud was acquired in a furniture-less state with completely empty rooms, all objects that are not represented in the ground truth data were manually removed from the point cloud and the triangle meshes acquired with the HoloLens. The floor was also removed, as it was hard to manually separate it from furniture objects. The evaluation against the TLS ground truth data was then conducted by assigning each point (respectively vertex in a triangle mesh) the Euclidean distance value to the nearest point of the ground truth data. Again, the software Cloud Compare [32] was used for this analysis.

## 3. Results

In this section, we present the results of the different experiments detailed in Section 2. First, in Section 3.1, we present the results of the evaluation of the HoloLens depth sensing capabilities. Afterwards, the results of the tracking evaluation are presented in Section 3.2, while Section 3.3 concludes with the results of the evaluation of the overall system for the use-case of indoor mapping.

### 3.1. Depth Sensing

**Figure 7:**
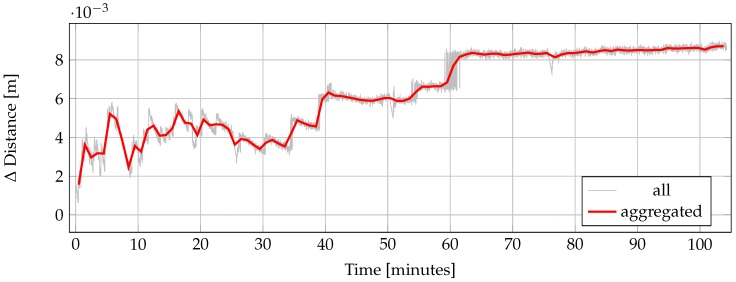
Change in distance measurement of the HoloLens range sensor relative to the start value over time during the warm-up process of the device. Vertical green lines indicate when the recording of range measurements had to be stopped and immediately restarted to prevent the device from switching to sleep mode.

In this section, we present the results of the evaluation of the HoloLens ToF range sensor as described in Section 2.2.1. Figure 7 shows the variation of the measured range value over time during the warm-up process of a completely cooled-down device relative to the first range measurement. In the first 40 min after the beginning of the measurements, the range value is subject to strong fluctuations in the range of few millimeters. Afterwards, from about 40 to 60 min, the range value remains more or less constant at a value of 6 mm above the initial value. Then, at a measurement time of about one hour, the measured value rises again by two to three millimeters accompanied by strong fluctuations. Afterwards it remains stable with a slightly increasing trend for the rest of the measurement.

**Figure 8:**
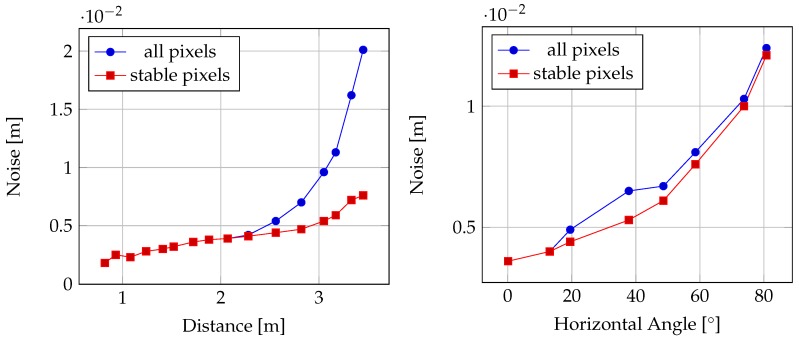
Noise of the distance measurements of the HoloLens ToF sensor against distance and inclination of a captured plane. Stable pixels are pixels that have range measurements in at least 75% of images.

Table 2 on the other hand shows the results of the investigation of sensor noise against distance and inclination of a captured plane. The given values for noise are calculated as mean standard deviations of the measured distance values over all pixels. The sensor noise is calculated once for all pixels on the captured wall surface and once only for those pixels that contain range values in at least 75% of images of a respective recording. The results are further visualized in Figure 8.

As Figure 8a shows, the sensor noise stays below 5 mm for measured distances smaller than about 2.5 m. From 2.5 m upwards, a rapid increase in noise is observable. This increase is mainly caused by pixels, that only sporadically return range measurements. When only considering stable pixels having range measurements in at least 75% of the recorded images, the increase in noise with distance proves less steep and stays below 1 cm for the whole measurable distance range.

In the case of the influence of surface inclination as visualized in Figure 8b however, sensor noise increases by approximately the same rate for only the stable pixels as in the case of considering all pixels. In both cases, noise remains below 5 mm for inclinations below 20°.

**Figure 9:**
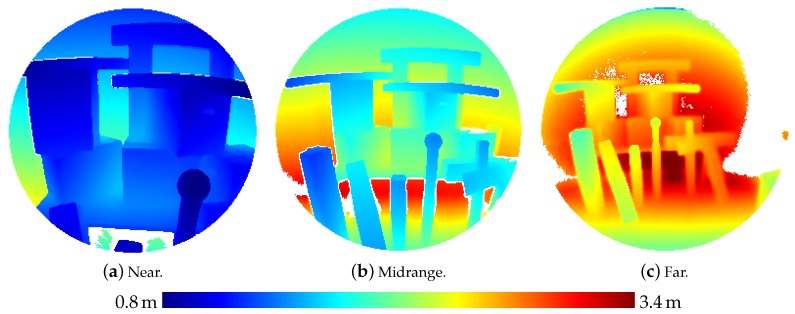
Depth images derived from the HoloLens ToF sensor for the three-dimensional scene depicted in Figure 5 captured from three different distances.

**Figure 10:**
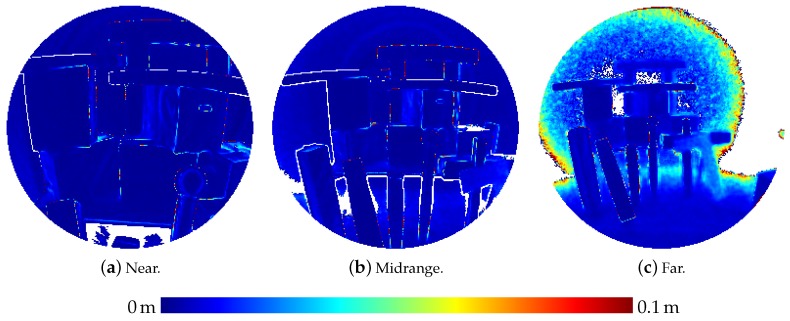
Noise of the range measurements of the HoloLens ToF sensor for the three-dimensional scene depicted in Figure 5 captured from three different distances.

**Figure 11:**
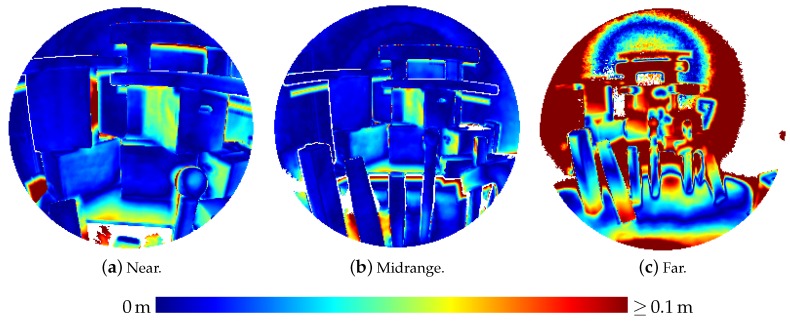
Accuracy of HoloLens range measurements evaluated against TLS ground truth data for the three-dimensional scene depicted in Figure 5 captured from three different distances.

**Figure 12:**
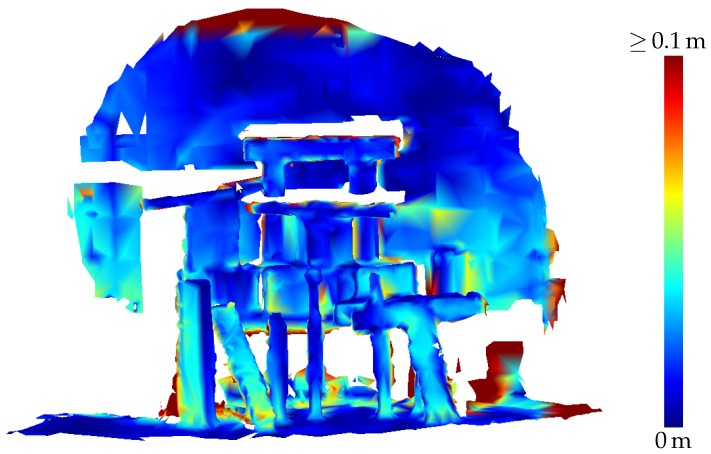
Accuracy of the HoloLens triangle mesh of the three-dimensional scene depicted in Figure 5 evaluated against TLS ground truth data.

Table 3 presents the results of the evaluation of the three-dimensional scene depicted in Figure 5. The results are also visualized in Figure 9 as depth images, while Figure 10 visualizes the noise and Figure 11 the accuracy of the range measurements evaluated against TLS ground truth data for all three distances the scene was captured from by the HoloLens range sensor (near, midrange and far). Furthermore, Figure 12 visualizes the accuracy of the HoloLens triangle mesh of the same scene evaluated against the TLS ground truth data.

### 3.2. Tracking

In this section, results of the evaluation of the HoloLens tracking system as detailed in Section 3.2 are presented.

The evaluation of eight trajectories against ground truth data determined by the motion capture system results in a mean ATE value of 1.9 ± 0.4 cm and a mean RPE value quantifying drift per second of 1.6 ± 0.2 cm and 2.2 ± 0.3°. Seven similar trajectories recorded with covered range sensor resulted in a mean ATE of 1.3 ± 0.1 cm and a mean RPE of 1.6 ± 0.1 cm and 1.5 ± 0.3°.

**Figure 13:**
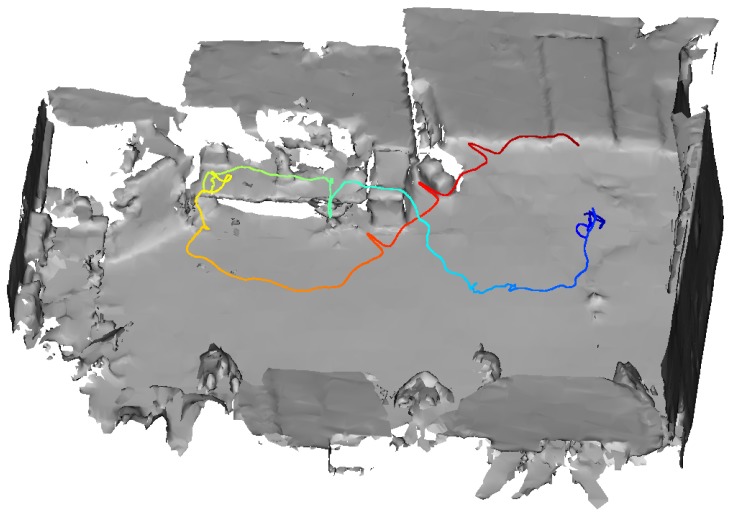
HoloLens trajectory in a room equipped with a Motion Capture System. The color represents time. The trajectory starts at blue and ends at red. The evaluation results in Figure 14 also refer to this trajectory. They are visualized in the same color range representing time.

**Figure 14:**
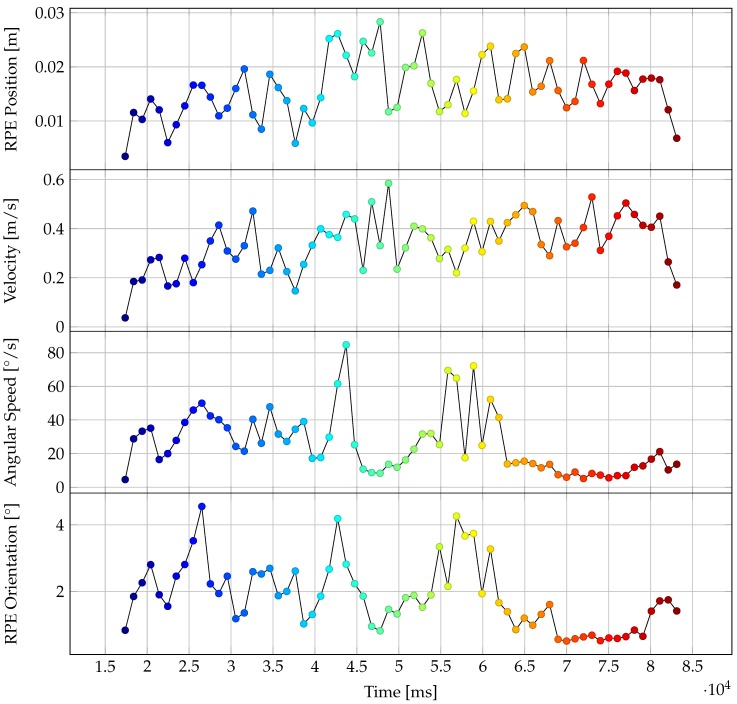
Velocity and RPE values over the course of the trajectory depicted in Figure 13. The color range is the same as the one from Figure 13, symbolizing the time going from blue to red.

One of the evaluated trajectories of the rigid body on the device as tracked by the HoloLens tracking system is depicted in Figure 13. This trajectory was recorded with non-covered range sensor. Figure 14 shows the associated velocity and RPE values over the course of the trajectory. The color range in both figures symbolizes time, going from blue to red.

**Figure 15:**
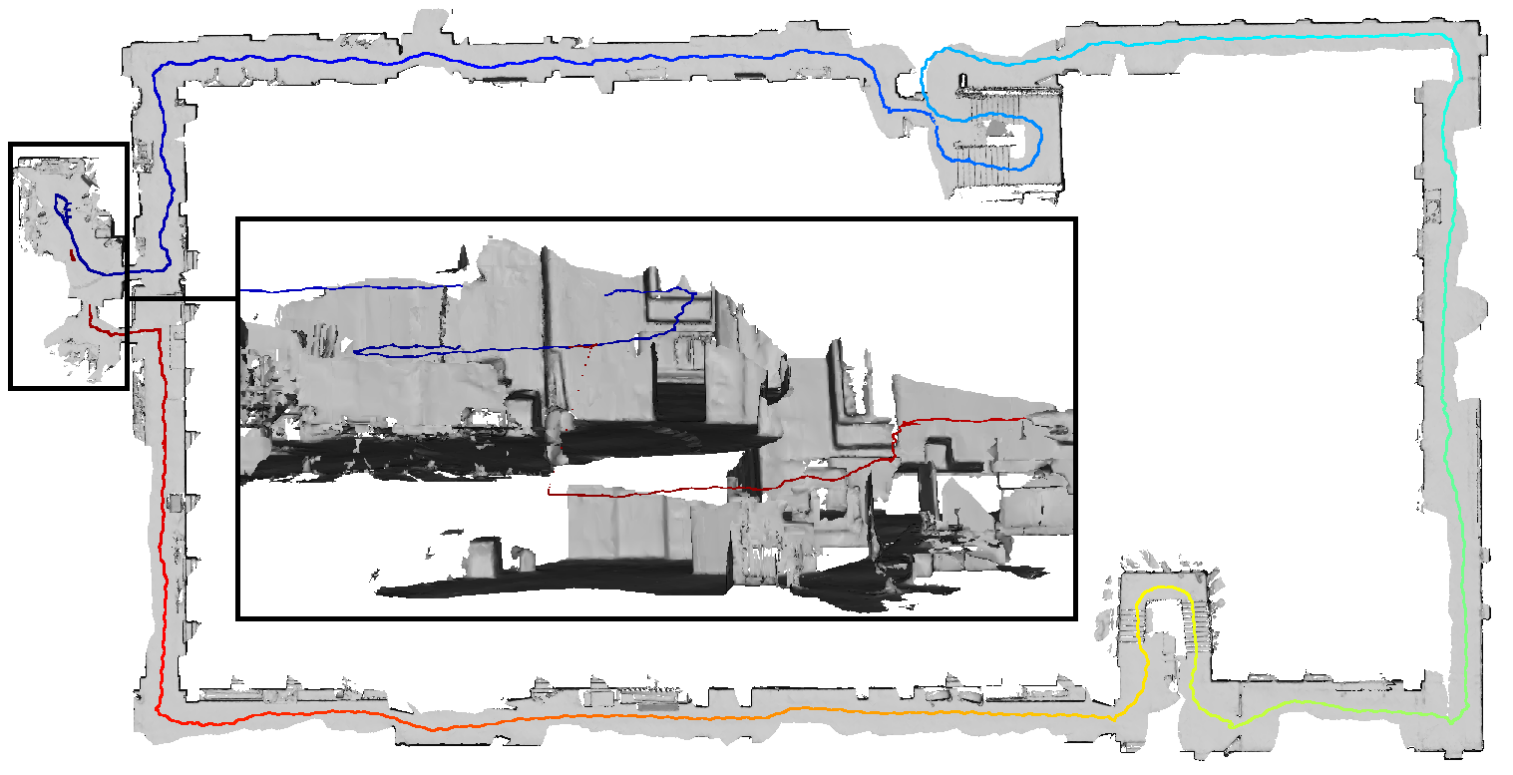
Closed trajectory of a total length of 287 m. The color represents time. The trajectory starts at blue and ends at red. The positional error caused by drift amounted to 2.39 m upon re-entering the room.

Finally, Figure 15 shows the result of the experiment to assess drift on large-scale trajectories described in Section 2.2.2. The travelled distance of the depicted trajectory totals to 287 m (including drift). The offset caused by drift upon re-entering the room amounts to 2.39 m.

### 3.3. Indoor Mapping

In this section, the results of the evaluation of the overall HoloLens system for the use-case of indoor mapping as described in Section 2.2.3 are presented.

**Figure 16:**
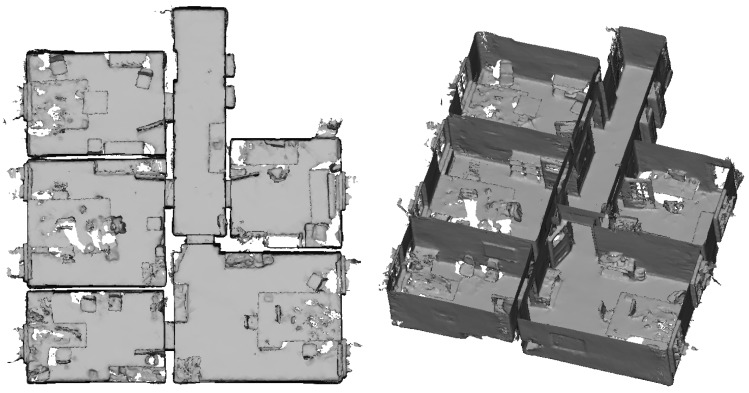
Triangle mesh of an indoor office environment captured by the HoloLens. The ceiling is removed for better visibility.

**Figure 17:**
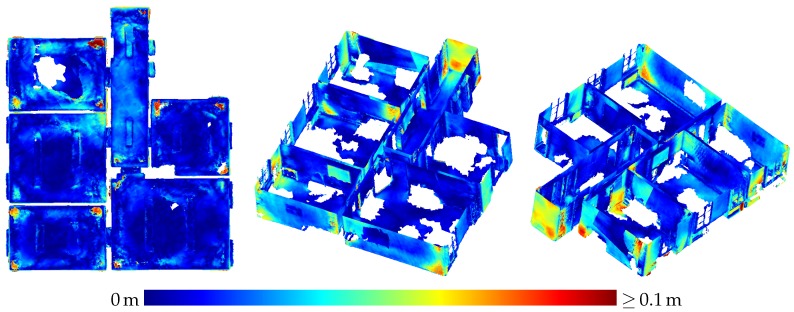
Accuracy of the HoloLens triangle mesh evaluated against the TLS ground truth. The registration was done with fixed scale. The mean distance to the ground truth amounts to 2.3 cm.

**Figure 18:**
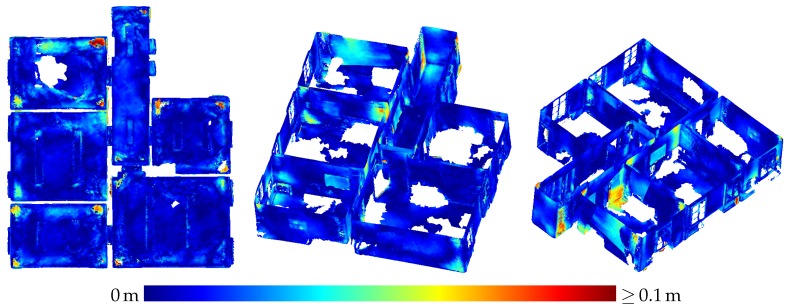
Accuracy of the HoloLens triangle mesh evaluated against the TLS ground truth. The scale factor was determined to 0.9938 by registration. The mean distance to the ground truth amounts to 1.7 cm.

Figure 16 depicts the triangle mesh captured of an indoor office environment consisting of five rooms and a small hallway. In Figure 17, the accuracy of the triangle mesh evaluated against TLS ground truth data is visualized. In this case, the mesh was registered on the ground truth data while keeping the scale fixed. The average accuracy of the complete mesh evaluated amounts to 2.3 cm. Figure 18 on the other hand depicts the accuracy evaluated against the same ground truth data resulting from a registration which also adapts the scale of the mesh. In this way, a scale factor of 0.9938 was determined, while the mean accuracy amounts to 1.7 cm.

**Figure 19:**
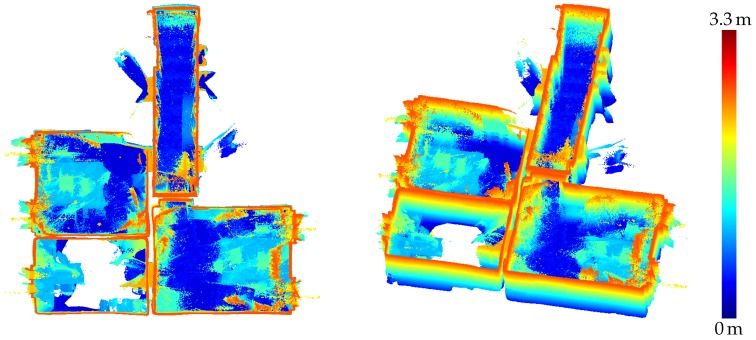
Point cloud of an indoor office environment captured by the HoloLens range sensor. The colors visualize point height with blue for the lowest and red for the highest points. The ceiling is removed for better visibility.

**Figure 20:**
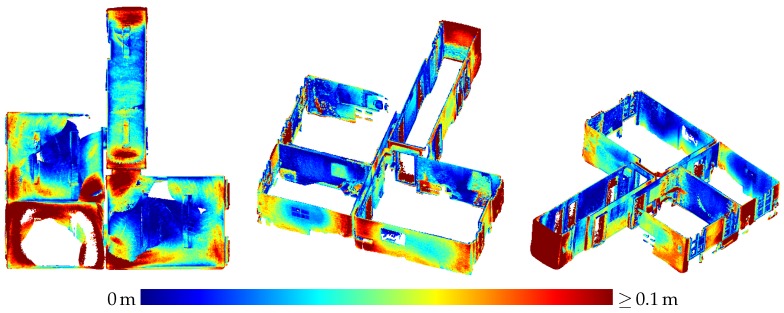
Accuracy of the HoloLens point cloud evaluated against the TLS ground truth. The registration was done with fixed scale. The mean distance to the ground truth amounts to 4.0 cm.

**Figure 21:**
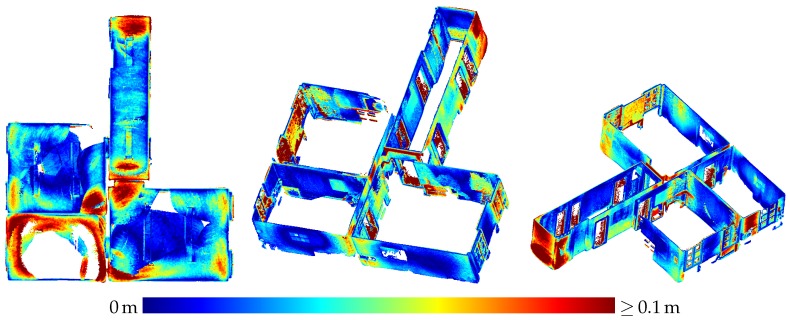
Accuracy of the HoloLens point cloud evaluated against the TLS ground truth. The scale factor was determined to 0.9887 by registration. The mean distance to the ground truth amounts to 2.4 cm.

Figure 19 shows a point cloud of a subset of three of the rooms and the hallway that was derived from range images captured by the HoloLens range camera and registered via the camera poses provided by the tracking system. The evaluation results for this point cloud are depicted in Figure 20 for fixed scale with a resulting mean accuracy of 4.0 cm and in Figure 21 for a scale factor of 0.9887 determined by registration and a resulting mean accuracy of 2.4 cm.

The indoor mapping process with the HoloLens took about 10 min for the capturing of the triangle mesh as depicted in Figure 16 with about 267,000 triangles and 5 MB in binary PLY format. The recording of the range images resulting in the point cloud depicted in Figure 20, however, took about 30 min resulting in 1763 range images. The creation of the resulting point cloud with about 70 million points and about 1 GB in binary PLY format took about 20 min. However, it has to be taken into account that we used a straight-forward implementation, that could further be optimized.

## 4. Discussion

In the following sections, the results of the experiments presented in Section 3 are discussed. We again start with the evaluation of depth sensing in Section 4.1, continue with discussing the results of the evaluation of the HoloLens tracking system in Section 4.2 and conclude with Section 4.3, where the results of the experiments dedicated to indoor mapping are discussed.

### 4.1. Depth Sensing

Regarding the influence of the warm-up process of the device on the accuracy of range measurements as presented in Figure 7, it can generally be recommended to let the device warm-up for at least one and a half hours before starting measurements with the HoloLens range camera, when precision is of importance. When using the device for indoor mapping tasks, this warm-up-induced drift in range measurements can potentially further increase drift effects caused by drift in tracking as reported in Section 3.2.

In the findings presented in Figure 8, noise in range measurements of up to 2 cm under unfavourable conditions (long distances, high inclination) were ascertained. However, in the context of indoor mapping, the influence of such effects cannot be easily assessed, as indoor mapping is generally a dynamic process affected by the movement of the user wearing the device through the environment to be mapped. In contrast, the findings presented in Section 3.1 apply to static situations, where a scene is captured from one fixed sensor position over a certain range of time. In the context of indoor mapping, it will rarely happen that a part of the scene is observed from only one position.

A user mapping his environment should take care to observe all surfaces of interest from a distance of at most about 2.2 m and from a not too steep angle. However, even if all relevant parts of the scene are captured by favourable sensor positions, there will always also arise range measurements that suffer from high noise caused by large distances or oblique angles. Raw indoor point clouds derived from HoloLens range images will thus always contain a high amount of noise as is apparent in Figure 19. This is further contributed to by errors in the sensor poses obtained from the tracking system.

Thus, HoloLens range measurements have to be further preprocessed e.g., by removing single points affected by high noise or whole range images affected by tracking errors to yield reasonable results. The triangle meshes provided by the HoloLens system, although produced in a black-box manner, can be regarded as the result of such a preprocessing. As shown in Table 3, the accuracy of the triangle mesh falls in the range between the range images captured under favourable conditions and the one suffering from a too large distance of the sensor to the scene. The mesh represents the accumulated knowledge the HoloLens system has of its environment after capturing range images from the three positions detailed in the table. However, the accuracy of the mesh as specified in Table 3 is lower as the accuracy of the range images captured from not too high distances. We suspect that this is caused by the reduction of spatial resolution due to the triangulation process.

**Figure 22:**
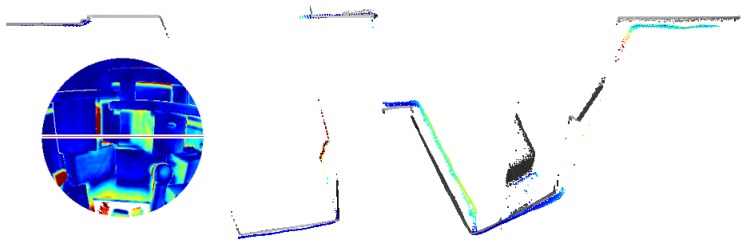
Cross section from Figure 11a corresponding to the red line on white background shown in the small range image in this figure. The large deviation of the range measurements on the left side of the right box from the ground truth (visualized in gray-scale) are possibly caused by multi-path effects.

Besides inaccuracy caused by sensor noise, there are also systematic effects degrading the accuracy of HoloLens depth sensing in some parts of the data. In Figure 11a, e.g., the left side of the box on the right is indicated by turquoise to yellow coloring to deviate quite strongly from the ground truth data. A horizontal cross section of this part is shown in Figure 22. This deviation could possibly be caused by multi-path effects. Other deviations possibly caused by multi-path effects include the upward bulging of triangle meshes occurring in corner situations on ceilings as indicated by red color in the top view visualizations on the left-hand side of Figure 17 and Figure 18.

### 4.2. Tracking

The evaluation results presented in Section 3.2 show that the HoloLens tracking system is capable of marker-less inside-out tracking in indoor environments with an accuracy of two centimeters or better. This is also supported by the apparent spatial stability of virtual objects as perceived by the user wearing the device. Figure 14 seems to imply a correlation of positional RPE values with velocity and rotational RPE with angular velocity over the course of the trajectory.

It is noteworthy, however, that our results seem to indicate a higher tracking accuracy when covering the range sensor. We assume that this is caused by the ToF range sensor of the HoloLens interfering with the motion capture system. In this case, both conducted experiments would not adequately assess the true HoloLens tracking accuracy as (i) in the case of the uncovered range sensor, the ground truth values would be distorted and (ii) in the case of the covered range camera, the system would not be evaluated in its usual working mode. In any case, the presented results can be regarded as a lower bound for the quality of the HoloLens tracking system, if we do not assume that using the range sensor degrades tracking quality.

Anyhow, even quite small drift effects as those observed in room-scale trajectories accumulate with travelled distance as shown in Figure 15. In situations as the one depicted, loop closure is detected by the HoloLens system after re-entering the room and the position of the device is corrected accordingly. The triangle meshes however are only corrected locally in the direct surrounding of the place of the detected loop closure by removing falsely positioned meshes that do not correspond to physical structures when they get in the field-of-view of the range sensor.

### 4.3. Indoor Mapping

Contrary to the large displacements of adjacent rooms reported by Hübner et al. [13], no displacements of such a magnitude were noticed in the indoor triangle mesh presented in Section 3.3. Only the wall between the upper and middle room on the left-hand side in Figure 16 shows a slight narrowing towards the room corners which could also be caused by multi-path effects as is probably the case with the outward bulging of ceilings in room corners visible as red spots in some room corners in the top view visualizations in Figure 17 and Figure 18.

The difference between our mesh and the one presented by Hübner et al. [13] is, that in our case, the indoor office environment contains furniture, while, in the other case, the rooms were completely devoid of any objects. We suspect that the large deviations between the individual rooms were maybe caused by this furniture-less state, the white texture-less walls causing a deterioration in tracking performance. As, in our case, the ground truth data also did not contain furniture, all parts of the triangle mesh representing objects not present in the ground truth data had to be removed manually.

Although the evaluation still establishes a significant scale factor, its impact on the accuracy of the triangle mesh is by no means as strong as the case reported by Hübner et al. [13]. With a mean accuracy of 1.7 cm for corrected scale and 2.3 cm for the original scale of the triangle mesh, these results demonstrate the high potential of the HoloLens for the use-case of indoor mapping.

Large-scale drifts in tracking as discussed in Section 4.2 however still prove an obstacle. In these cases, it would be necessary to distribute a detected offset over the whole trajectory and its attached meshes in the event of loop closure detection. Corrections like this are not taken into consideration for the HoloLens as it is not needed for its actual use-case as an augmented reality device where only the correctness of the triangle mesh in the direct vicinity of the user is of importance.

The evaluation of the point cloud of a subset of four rooms of the same indoor environment derived from range images of the HoloLens ToF camera resulted in an accuracy of 2.4 cm for corrected scale and 4.0 cm for the original scale.

This accuracy is lower than the resulting accuracy of the triangle meshes of the same environment whereas the evaluation of the scene presented in Section 3.1 resulted in the triangle meshes showing a lower accuracy than range measurements captured under suitable conditions. In this case, however, the sensor was capturing the scene in a static setting for a certain duration whereas, here, it was constantly moving through the environment with the user. Thus, as already discussed in Section 4.1, every part of the mapped indoor environment can be expected to not only have been captured in favorable constellations, but also from high distances or steep angles. Furthermore, inaccuracies in the tracking of the device pose propagate to the global position of points resulting from range images. The resulting point clouds are thus characterized by a huge amount of noise, as can be seen in Figure 19. Besides, some parts of the indoor environment were only ever captured under unfavorable conditions by the range sensor. For example, in the case of the lower left room depicted in the top view visualization in Figure 20 and Figure 21, the operator mapping the environment forgot to look upwards to the ceiling. The ceiling surface in this room was thus only captured partially and only under oblique angles, which results in low accuracy in the respective part of the point cloud.

## 5. Conclusions

In this work, we provided a thorough evaluation of the Microsoft HoloLens regarding its adequacy for the use-case of indoor mapping. After a brief survey of the different camera sensors the device is equipped with, we independently evaluated the performance of its depth sensing and tracking system. Subsequently, we evaluated the complete system in respect of the task of mapping indoor environments.

While we demonstrated the potential of the HoloLens as an off-the-shelf tool for indoor mapping, we also highlighted its shortcomings. It however has to be remembered that the HoloLens was not primarily designed as an indoor mapping device. It rather is a mobile augmented reality headset. Thus, its capabilities in capturing the geometry of its surrounding are geared towards the needs of an AR device, where typically only the direct vicinity of the user that is to be augmented with virtual content needs to be consistently known. Large-scale drift in tracking and the deviations in the captured meshes caused by it are not a problem from the viewpoint of augmented reality, as the user only ever perceives his current vicinity which is captured sufficiently consistent to allow for virtual content to realistically interact with the physical environment.

Nevertheless, the HoloLens as an off-the-shelf, rather low-cost device that is easy to use still holds great promise for effortlessly capturing the geometric structure of large indoor environments.

Regarding potential future work on the evaluation of the HoloLens or similar sensor systems, the investigations presented in this paper can certainly be further extended and deepened. The evaluation of the range sensor should particularly be extended by a wider variety of test objects and scenarios. For instance, examining further test geometries and constellations could enable further insight on the behaviour of multi-path effects. In addition, investigating the influence of different surface materials holds potential for further research. Furthermore, the recently introduced second version of the HoloLens should also be comparatively examined regarding its potential for the use-case of indoor mapping.

## Figures and Tables

**Table 1:** HoloLens camera sensors and their characteristics as indicated by the Microsoft Windows 10 SDK for the device.

Camera	Type	Field-of-View [°]	Image Size [pixels]	Effective Pixels [%]	Frame Rate [fps]	Data Type
**Photo Video**	Color	40 × 25	1408 × 792 1344 × 756 1280 × 720 896 × 504	100	30	BGRA8 ^a^
**Long Throw**	Depth	60 × 54	448 × 450	24 ^b^	1 ^c^	Gray16
**Long Throw**	Intensity	60 × 54	448 × 450	24 ^b^	1^c^	Gray8
**Short Throw**	Depth	78 × 77	448 × 450	71 ^b^	15	Gray16
**Short Throw**	Intensity	78 × 77	448 × 450	71 ^b^	15	Gray8
**4 × Tracking**	Grayscale ^d^	60 × 50	160 × 480 ^d^	400 ^d^	30	BGRA8 ^d^

^a^ The alpha channel contains a constant value of 1.0. ^b^ Only a fraction of the image actually contains values (see Figure 3). ^c^ The SDK reports a frame rate of 3 fps, but an actual frame rate of 1 fps was observed. ^d^ The system returns a 160 × 480 4-channel image, which actually represents a 640 × 480 grayscale image spread line-wise over all four channels.

**Table 2:** Noise of the distance measurements of the HoloLens ToF sensor against distance and inclination of a captured plane. Stable pixels are pixels that have range measurements in at least 75% of images. Part (a): variation of distance; part (b): variation of inclination.

	Horizontal Angle [°]	Vertical Angle [°]	Depth [m]	Noise [m]	Noise in Stable Pixels [m]	Fraction of Stable Pixels [%]
(a)	0.5	0.6	0.82	0.0018	0.0018	100
	0.2	5.9	0.93	0.0025	0.0025	100
	−1.9	4.3	1.08	0.0023	0.0023	100
	−3.1	4.7	1.24	0.0028	0.0028	100
	2.5	3.8	1.41	0.0030	0.0030	100
	1.8	3.9	1.52	0.0032	0.0032	100
	0.1	4.8	1.72	0.0036	0.0036	100
	−2.4	5.3	1.88	0.0038	0.0038	100
	−0.9	4.7	2.07	0.0039	0.0039	100
	1.8	5.0	2.28	0.0042	0.0041	100
	3.9	5.0	2.56	0.0054	0.0044	95
	3.8	4.6	2.82	0.0070	0.0047	84
	3.8	4.5	3.05	0.0096	0.0054	74
	0.4	4.5	3.17	0.0113	0.0059	69
	5.6	5.2	3.33	0.0162	0.0072	51
	5.5	5.1	3.45	0.0201	0.0076	18
(b)	0.1	4.8	1.72	0.0036	0.0036	100
	13.1	5.8	1.84	0.0040	0.0040	100
	19.5	8.6	1.94	0.0049	0.0044	98
	37.8	−2.0	1.89	0.0065	0.0053	96
	48.6	−2.0	1.84	0.0067	0.0061	97
	58.6	1.1	1.69	0.0081	0.0076	97
	73.8	−8.4	1.55	0.0103	0.0100	98
	80.8	−29.6	1.38	0.0124	0.0121	99

**Table 3:** Evaluation of a three-dimensional scene as depicted in Figure 5 captured by the HoloLens ToF sensor from three different distances regarding noise and accuracy against TLS ground truth. Additionally, a HoloLens triangle mesh of the scene is also evaluated against TLS ground truth. For visual representation of the results, see Figure 9, Figure 10, Figure 11 and Figure 12.

Recording	Mean Depth [m]	Mean Noise [m]	Mean Noise in Stable Pixels [m]	Mean Distance to Ground Truth [m]
**Near**	1.30	0.0043	0.0042	0.0188
**Midrange**	2.02	0.0062	0.0060	0.0138
**Far**	2.64	0.0190	0.0104	0.0939
**Mesh**	—	—	—	0.0458
